# Transparent and Multi‐Foldable Nanocellulose Paper Microsupercapacitors

**DOI:** 10.1002/advs.202203720

**Published:** 2022-10-18

**Authors:** Sang‐Woo Kim, Kwon‐Hyung Lee, Yong‐Hyeok Lee, Won‐Jae Youe, Jae‐Gyoung Gwon, Sang‐Young Lee

**Affiliations:** ^1^ Department of Energy and Chemical Engineering Ulsan National Institute of Science and Technology (UNIST) UNIST‐gil 50, Eonyang‐eup, Ulju‐gun Ulsan 44919 Republic of Korea; ^2^ Department of Chemical and Biomolecular Engineering Yonsei University 50, Yonsei‐ro, Seodaemun‐gu Seoul 03772 Republic of Korea; ^3^ Department of Forest Products National Institute of Forest Science Seoul 02455 Republic of Korea

**Keywords:** biopolymer, foldable electronics, nanocellulose, supercapacitors, transparent power source

## Abstract

Despite the ever‐increasing demand for transparent power sources in wireless optoelectronics, most of them have still relied on synthetic chemicals, thus limiting their versatile applications. Here, a class of transparent nanocellulose paper microsupercapacitors (TNP‐MSCs) as a beyond‐synthetic‐material strategy is demonstrated. Onto semi‐interpenetrating polymer network‐structured, thiol‐modified transparent nanocellulose paper, a thin layer of silver nanowire and a conducting polymer (chosen as a pseudocapacitive electrode material) are consecutively introduced through microscale‐patterned masks (which are fabricated by electrohydrodynamic jet printing) to produce a transparent conductive electrode (TNP‐TCE) with planar interdigitated structure. This TNP‐TCE, in combination with solid‐state gel electrolytes, enables on‐demand (in‐series/in‐parallel) cell configurations in a single body of TNP‐MSC. Driven by this structural uniqueness and scalable microfabrication, the TNP‐MSC exhibits improvements in optical transparency (*T* = 85%), areal capacitance (0.24 mF cm^−2^), controllable voltage (7.2 V per cell), and mechanical flexibility (origami airplane), which exceed those of previously reported transparent MSCs based on synthetic chemicals.

## Graphical abstract

Transparent nanocellulose paper microsupercapacitos (TNP‐MSCs) as a natural material strategy are presented to overcome the challenges of synthetic chemicals‐based transparent power sources. The TNP‐MSCs with a microscale planar interdigitated structure enable the on‐demand cell configurations with tunable voltages and capacitances in a single body, and exhibit superior optical transparency and mechanical deformability, which outperform those of previously reported transparent MSCs.

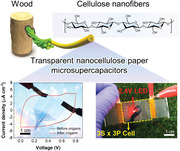

## 1 Introduction

The ongoing surge in the demand for smart portable electronics, With the emergence of the wireless optoelectronics era,^[^ ^1^, ^2^, ^3^, ^4^, ^5^ ^]^ transparent displays, smart windows/glasses, and touch screens have attracted widespread attention, which thus spurs the relentless pursuit of high‐performance transparent power sources to enable the ubiquitous access and portable use of the optoelectronics. A key component of transparent power sources is transparent conductive electrodes (TCEs). Previous studies have focused on tin‐doped indium oxides (ITO) as a core material of TCEs. However, the inherent brittleness and fragility of ITO‐based TCEs have limited their versatile applications.^[^ ^6^, ^7^, ^8^ ^]^

To resolve this problem, several alternatives to ITO have been explored. In particular, transparent polymeric substrates, including polyethylene terephthalate (PET) and polydimethylsiloxane (PDMS), have attracted significant attention.^[^ ^9^, ^10^, ^11^, ^12^ ^]^ However, most polymeric substrates are hydrophobic and not mechanically robust, which results in weak interfacial adhesion with adjacent conductive layers upon mechanical deformation.^[^ ^2^, ^13^ ^]^ Moreover, the thermal intolerance of polymeric substrates owing to their low glass transition temperatures (mostly below 100 °C) and high coefficients of thermal expansion (CTE) results in the loss of their dimensional integrity and optical transparency upon exposure to high‐temperature manufacturing/operating environments.^[^ ^14^ ^]^

In addition to the material issues described above, another critical challenge facing the TCEs lies in fabrication techniques. For example, scaling down of electrode resolutions below visible wavelength^[^ ^15^, ^16^ ^]^ is an essential requirement for TCE fabrication, which thus pushes us to pay more attention to electrode pattern design and associated microfabrication methods. Many previous studies^[^ ^17^, ^18^, ^19^ ^]^ on TCEs have used complex and cost‐consuming fabrication techniques, such as photolithography/selective etching. In addition, the difficulties in diversifying the structure of TCEs and widening their operation voltages has restricted their further application. Therefore, to enable practical TCEs with electrochemical reliability and application versatility, facile and scalable microfabrication techniques should be developed, in addition to overcoming the material limitations.

Here, to address the aforementioned longstanding challenges of synthetic chemicals‐based TCEs, we present a class of transparent nanocellulose paper microsupercapacitors (TNP‐MSCs) as a natural material strategy. Cellulose nanofibers (CNFs) have attracted increasing attention in various application fields owing to their natural abundance, environmental friendliness, recyclability, and facile functionalization.^[^ ^20^, ^21^, ^22^ ^]^ Among several CNFs, 2,2,6,6‐tetramethylpiperidin‐1‐oxyl‐oxidized CNFs (TEMPO‐CNFs) have been investigated as a transparent material for optoelectronics.^[^ ^23^, ^24^ ^]^ However, their mechanical stiffness and susceptibility to fracture upon mechanical deformation^[^ ^25^, ^26^ ^]^ have limited their application in state‐of‐the‐art flexible optoelectronics. To overcome the limitations of TEMPO‐CNFs, we proposed a semi‐interpenetrating polymer network (semi‐IPN)‐structured TNP concept, consisting of TEMPO‐CNF and crosslinked N‐hydroxyethyl acrylamide (HEAA). Subsequently, a semi‐IPN TNP‐based transparent conductive film (TNP‐TCF) was prepared by subjecting the semi‐IPN TNP substrate to thiol (‐SH) treatment, after which silver nanowire (AgNW) conductive layers were introduced. The TNP‐TCF exhibits superior mechanical flexibility and thermal tolerance compared to previously reported typical PET‐TCF.

As a proof‐of‐concept for this transparent nanocellulose paper‐based approach, MSCs are selected owing to their power capability, cyclability, material versatility, and efficient space utilization.^[^ ^27^, ^28^ ^]^ Moreover, MSCs have been extensively investigated as a promising power source for flexible/wearable electronics.^[^ ^29^, ^30^, ^31^ ^]^ It is essential to rationally design the underlying structure of MSCs to prevent unwanted loss in their optical transparency upon the introduction of MSC components, such as electrodes and electrolytes. To resolve this issue, ultraviolet (UV)‐cured polymeric masks with microscale patterns are introduced on the TNP‐TCF using electrohydrodynamic (EHD) jet printing rather than traditional photolithography methods. EHD jet printing, which is a state‐of‐the‐art drop‐on‐demand printing, has recently garnered substantial attention as a facile, high‐throughput, and high‐resolution microfabrication technique.^[^ ^32^, ^33^ ^]^ Onto the EHD jet‐printed micropatterned mask, poly(3,4‐ethylene dioxythiophene):(styrene sulfonate) (PEDOT:PSS),^[^ ^34^, ^35^ ^]^ which is selected as a pseudocapacitive electrode active material for the MSCs, is air‐sprayed to fabricate a nanocellulose‐based transparent conductive electrode (TNP‐TCE) with a microscale planar interdigitated structure. Combined with solid‐state gel electrolytes that can ionically isolate MSC unit cells, this TNP‐TCE enables on‐demand (in‐series/in‐parallel) cell configurations with tunable voltages and capacitances in a single body of TNP‐MSC. In addition, a hydrophobic silane‐treated TNP is prepared as a water‐proof packaging substance to fabricate nanocellulose paper MSCs.

Owing to the TNP‐based material uniqueness and the EHD‐driven scalable microfabrication, the resulting TNP‐MSC achieves improvements in optical transparency (transmittance (*T*) = 85% at 550 nm wavelength), customizable voltage (7.2 V per cell), and mechanical deformability (bending, multiple folding, and even origami airplane), which outperform those of previously reported transparent MSCs based on synthetic chemicals.

## 2 Results

### 2.1 Preparation of Semi‐IPN TNP as a Beyond‐synthetic‐Material Strategy

**Figure 1:**
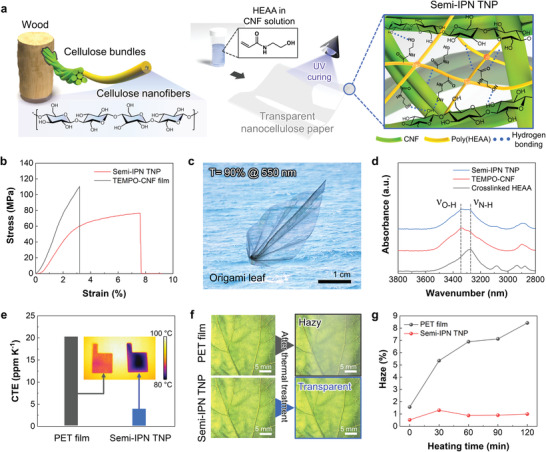
Preparation and characterization of semi‐IPN TNP. a) Schematic illustration of the synthesis of semi‐IPN TNP that features the intermolecular hydrogen bonding between the TEMPO‐CNF and UV‐crosslinked HEAA. b) Stress‐strain curves of the semi‐IPN TNP and TEMPO‐CNF film. c) Photograph of the origami‐folded semi‐IPN TNP‐based transparent paper leaf. d) FT‐IR peaks (3000–3600 cm^−1^) of the hydroxyl groups of the semi‐IPN TNP (versus TEMPO‐CNF and crosslinked HEAA). e) Comparison of the CTE of the semi‐IPN TNP and PET film (control). f) Photographs of the semi‐IPN TNP and PET film (control) after exposure to heat at 140 °C for 2 h. g) Change in the optical haze of the semi‐IPN TNP and PET film (control) after heating at 140 °C as a function of the heating time.

To fabricate the TNP‐MSCs, first, we prepared a semi‐IPN structured TNP (**Figure** **1**a), in which a semi‐IPN design was adopted to improve the mechanical flexibility of a pristine TEMPO‐CNF film without impairing its optical transparency. The TEMPO‐CNF was mixed with HEAA having a refractive index of 1.51 which is close to that (1.55) of TEMPO‐CNF.^[^ ^36^ ^]^ The HEAA (containing 1.25 wt.% N,N’‐methylenebis (acrylamide) as a crosslinker) in the TEMPO‐CNF/HEAA mixture was UV‐crosslinked to obtain a transparent semi‐IPN TNP (transmittance (*T*) = 90% at 550 nm wavelength, which is comparable to that of the pristine TEMPO‐CNF film (90%)). The UV irradiation‐driven crosslinking was verified by monitoring the change in the Fourier‐transform infrared spectroscopy (FT‐IR) peaks assigned to acrylic C=C bonds (1610–1625 cm^−1^)^[^ ^37^, ^38^ ^]^ of HEAA (Figure S1).

The tensile properties of the semi‐IPN TNP were quantitatively examined (Figure 1b). The mechanical toughness of the semi‐IPN TNP was significantly higher that of the TEMPO‐CNF film. This result was confirmed by analyzing the fractured structure of the semi‐IPN TNP after the tensile test (Figure S2, Supporting Information). Compared to the fractured TEMPO‐CNF film, which showed relatively smooth and flat edges, the fractured semi‐IPN TNP exhibited rough and irregular edges. This result was consistent with those of typical ductile polymers,^[^ ^39^ ^]^ which exhibit a brittle‐to‐ductile transition behavior. Furthermore, the semi‐IPN TNP maintained its structural integrity under various mechanical deformation modes, such as bending, winding, and multiple folding (Figure S3, Supporting Information), whereas the TEMPO‐CNF film was easily fractured into several pieces. In addition, the mechanical flexibility of the semi‐IPN TNP was sufficient to enable its utilization for the fabrication of a transparent paper leaf via origami folding (Figure 1c). This substantial enhancement in the mechanical flexibility of the semi‐IPN TNP was attributed to the intermolecular hydrogen bonding^[^ ^40^, ^41^ ^]^ between the TEMPO‐CNF and UV‐cross‐linked HEAA, which is conceptually illustrated at the right side of Figure 1a. Furthermore, compared to those of the pristine TEMPO‐CNF and crosslinked HEAA (Figure 1d), the semi‐IPN TNP exhibited broadened characteristic FT‐IR peaks (3000–3600 cm^−1^)^[^ ^42^, ^43^ ^]^ of hydroxyl (OH) groups, verifying the formation of intermolecular hydrogen bonds in the semi‐IPN TNP.

Flexible optoelectronics are generally subjected to high‐temperature conditions during manufacturing processes and operations. Particularly, the difference in the CTE values of the constituent layers of electronic devices may induce undesired stress evolution at their interfaces, resulting in unwanted mechanical distortion or delamination.^[^ ^44^, ^45^ ^]^ The semi‐IPN TNP showed a significantly lower CTE value (3.8 ppm K^−1^) compared to that of a previously reported PET film (25 ppm K^−1^) which was selected as the control sample (Figure 1e). Typically, a CTE value of less than 20 ppm K^−1^ is required for the substrates of thin‐film electronic devices.^[^ ^46^, ^47^ ^]^ The semi‐IPN TNP and PET films were exposed to a high temperature of 140 °C for 2 h and then the change in their optical transparency was examined (Figure 1f). This heat treatment had no significant effect on the optical transparency of the semi‐IPN TNP. In contrast, unwanted byproducts, which may stem from cyclic oligomers,^[^ ^48^ ^]^ were randomly dispersed on the PET film surface (Figure S4, Supporting Information), resulting in optical haze due to light scattering. Next, the effect of the heating time at 140 °C on the optical haze (at 550 nm wavelength) of the two films was quantitatively examined (Figure 1g). The optical haze of the PET film increased from 1.5% to 8.4% with the elapsed time. In contrast, the change in the haze (below 1%) of the semi‐IPN TNP was negligible. This superior thermal stability of the semi‐IPN TNP was verified by performing a dynamic mechanical analysis (DMA). Over a wide range of temperature, the storage modulus of the semi‐IPN TNP was steadily maintained at ≈15 GPa and there was a negligible change in tan *δ*, whereas the PET film showed a notable change in both the storage modulus and tan *δ* at *T*_g_ (≈100 °C) (Figure S5, Supporting Information).

In addition to the aforementioned thermal tolerance, the semi‐IPN TNP should present an oxygen barrier property to enable its application in substrates and packaging materials for transparent power sources. Compared to the PET film (7.9 mL mm m^−2^ day^−1^ kPa^−1^), the semi‐IPN TNP showed an extremely low oxygen permeability (0.75 mL mm m^−2^ day^−1^ kPa^−1^) (Figure S6, Supporting Information). These superior physicochemical properties of the semi‐IPN TNP demonstrated its potential as a new transparent substrate that can outperform conventional PET films.

### 2.2 Performance Superiority of TNP‐TCF over PET‐TCF

**Figure 2:**
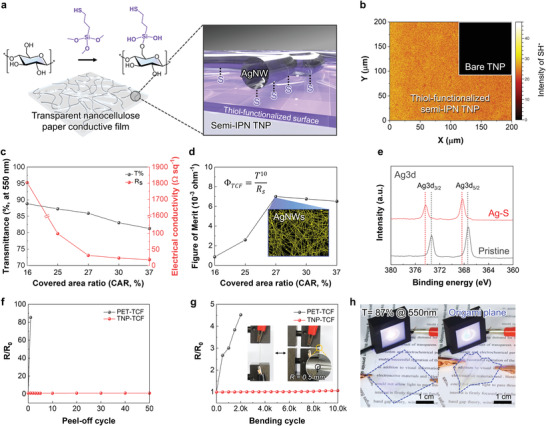
Performance superiority of TNP‐TCF over PET‐TCF. a) Schematic illustration of the preparation of thiol‐functionalized semi‐IPN TNP via a polycondensation reaction between hydroxyl (from the CNF) and thiol groups (from the MPTMS). b) TOF‐SIMS image of SH^−^ fragments in the thiol‐functionalized semi‐IPN TNP. c) Optical transmittance at 550 nm and electrical conductivity of the TNP‐TCFs as a function of the CAR. d) Figure of merit (*Φ*_TCF_) of the TNP‐TCFs as a function of the CAR. e) Ag 3d XPS spectra of the AgNWs in the TNP‐TCF (versus pristine AgNWs). f) Change in the electrical resistance of the TNP‐TCF (vs. PET‐TCF (control)) as a function of the peel‐off cycle at a peel‐off speed of 5.0 mm min^−1^. g) Change in the electrical resistance of the TNP‐TCF (vs. PET‐TCF (control)) as a function of the bending cycle (*R*_b_ = 0.5 mm and deformation rate = 100 mm min^−1^). Insets are photographs showing the physical appearance of the samples during the bending test. (h) Photographs showing the operation of a LED by the TNP‐TCF, which acted as an electrical conducting substrate under flat and origami airplane‐shaped states.

The AgNWs conductive layers were introduced on top of the as‐prepared semi‐IPN TNP substrate using an air‐spraying coater to prepare a semi‐IPN TNP‐based TCF (denoted as TNP‐TCF). It is essential to ensure strong interfacial adhesion between conductive layers and transparent substrates to enable flexible TCFs. Inspired by the Ag−S bonding theory,^[^ ^49^, ^50^ ^]^ we modified the semi‐IPN TNP substrate using thiol groups that can form intermolecular bonding with adjacent AgNWs conductive layers. The semi‐IPN TNP was reacted with 3‐mercaptopropyltrimethoxysilane (MPTMS) via a polycondensation reaction between hydroxyl (–OH, from the CNF) and thiol (–SH, from the MPTMS) groups to obtain thiol‐functionalized semi‐IPN TNP (**Figure** **2**a). A time‐of‐flight secondary ion mass spectroscopy (TOF‐SIMS) 2D mapping image revealed the uniform distribution of the thiol groups on the semi‐IPN TNP substrate (Figure 2b).

The morphology of the AgNWs conductive layer formed on the thiol‐functionalized semi‐IPN TNP substrate was tuned by adjusting the spraying pressure. With an increase in the pressure from 20 to 50 kPa, the number of AgNWs gradually increased over a wide area of the thiol‐functionalized semi‐IPN TNP substrate (Figure S7a, Supporting Information). The areal number density of the AgNWs was quantitatively estimated using digital image processing (Figure S7b, Supporting Information).^[^ ^51^ ^]^ The covered area ratio (CAR) increased from 16% to 37% with an increase in the spraying pressure, and the photographs of the obtained TNP‐TCFs (area = 2.0 × 2.0 cm^2^) as a function of the CAR are shown in Figure S8, Supporting Information. The optical transparency and electrical conductivity of the TNP‐TCFs were examined as a function of the CAR (Figure 2c). The electrical conductivity of the TNP‐TCFs increased with an increase in the CAR, whereas the optical transparency (measured at a wavelength of 550 nm) decreased. The optimal CAR value of the TNP‐TCFs was determined by estimating the figure of merit (*Φ*_TCF_), which can be expressed using the Haacke's equation^[^ ^52^ ^]^:

(1) $$\Phi_{TCF}=\frac{T^{10}}{R_{s}}=\sigma \;t\;\exp\left(-10\alpha t\right)$$

where *T* is the transmittance at 550 nm wavelength, *R*_S_ is the sheet resistance (Ω sq^−1^), *σ* is the electrical conductivity (Ω^−1^ cm^−1^), *t* is the film thickness (cm), and *α* is the optical absorption coefficient (cm^−1^). Figure 2d shows the *Φ*_TCF_ as a function of the CAR. The maximum *Φ*_TCF_ value (7.2 × 10^−3^ Ω^−1^) was achieved at a CAR of 27%. The morphology of the AgNWs at the optimal CAR (27%) is shown in an inset of Figure 2d.

The Ag–S bonding of the TNP‐TCF was examined using X‐ray photoelectron spectroscopy (XPS, Figure 2e). The binding energies of Ag 3d_3/2_ (at 373.5 eV) and Ag 3d_5/2_ (at 373.5 eV) of the pristine AgNWs shifted to higher values, indicating the intermolecular interaction^[^ ^53^, ^54^ ^]^ between the AgNWs and thiol groups of the TNP‐TCF. Compared to the control sample (PET‐TCF) which almost completely lost its AgNWs layer just after one peel‐off cycle (Figure S9, Supporting Information), the TNP‐TCF stably maintained its AgNWs layer after 50 peel‐off cycles owing to the Ag‐S bonding. This result was quantitatively confirmed by measuring the electrical resistance of the samples as a function of the peel‐off cycle (Figure 2f). An increase in the peel‐off cycle had a negligible effect on the electrical resistance of the TNP‐TCF, whereas the electrical resistance of the PET‐TCF increased abruptly.

This strong intermolecular bonding between the AgNWs layer and thiol‐functionalized semi‐IPN TNP substrate can positively affect the mechanical flexibility of the resulting TNP‐TCF. Figure 2g shows the change in the electrical resistance of the TNP‐TCF as a function of the bending cycle. The electrical resistance of the TNP‐TCF remained almost unchanged even after 10 000 bending cycles. In contrast, the PET‐TCF showed a sharp increase in electrical resistance and became electrically inert just after 1000 cycles. The enhanced mechanical bending of the TNP‐TCF was further highlighted by making a comparison with those of previously reported TCFs based on synthetic polymer substrates, such as PET and PDMS (Table S1, Supporting Information). The TNP‐TCF (T = 87% and *R*_S_ = 31 Ω sq^−1^; size = 3.0 × 3.0 cm^2^) was folded multiple times into an origami airplane to highlight its application as an electrical conducting substrate for flexible electronics. The origami‐folded TNP‐TCF successfully operated an LED lamp without the loss of its optical transparency and electrical conductivity (Figure 2h). In contrast, the structural disruption and delamination of the AgNWs layer of PET‐TCF were observed upon its origami folding (Figure S10, Supporting Information). These results demonstrated the viability of the TNP‐TCF as a promising alternative to typical synthetic polymer‐based TCFs for transparent flexible electronics.

### 2.3 TNP‐TCE with a Microscale Planar Interdigitated Structure

**Figure 3:**
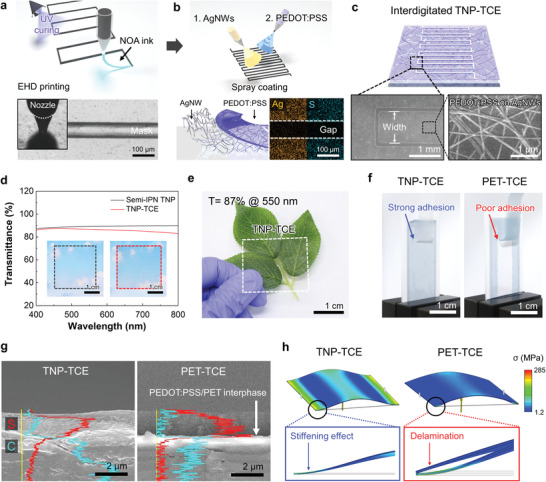
TNP‐TCEs with a microscale planar interdigitated structure. a,b) Schematic representation of the stepwise fabrication procedure (top) of the TNP‐TCE and its optical microscopy and EDS images (bottom). c) Schematic (top) and SEM images (bottom) of the TNP‐TCE. d) Optical transmittance of the semi‐IPN TNP and TNP‐TCE in the wavelength range of 400–800 nm (visible light). Insets are their photographs. e) Photograph of the TNP‐TCE showing its optical transparency. f) Peel‐off test (at a peel‐off speed of 5.0 mm min^−1^) results of the TNP‐TCE and PET‐TCE (control sample). g) Depth profiles of the carbon and sulfur elements in the TNP‐TCE and PET‐TCE (control sample) in the through‐thickness direction, with focus on the interface between the PEDOT:PSS (top layer) and substrates (bottom layer). h) FEM simulation of the stress and deformation of the TNP‐TCE and PET‐TCE (control sample).

To fabricate TNP‐TCE with a microscale planar interdigitated structure, UV‐curable mask ink (a mixture of NOA68T/SiO_2_ nanoparticle = 80/20 (w/w)) was EHD jet‐printed on the semi‐IPN TNP substrate (Movie S1, Supporting Information) and was then subjected to UV crosslinking to obtain a microscale mask with interdigitated array patterns (**Figure** **3**a). The system configuration of the EHD jet‐printing equipment is schematically illustrated in Figure S11, Supporting Information. The UV‐curable mask ink (i.e., before UV irradiation) showed a typical shear‐thinning fluid behavior,^[^ ^34^, ^55^ ^]^ which is suitable for EHD jet printing (Figure S12, Supporting Information). Subsequently, an AgNW ink and an electrode ink (PEDOT:PSS) were introduced sequentially on the semi‐IPN TNP substrate with the microscale patterned mask using the spray coating process. The TNP‐TCE with a microscale planar interdigitated PEDOT:PSS electrode layer was obtained after removing the micropatterned mask (Figure 3b). Energy dispersive X‐ray spectroscopy (EDS) elemental mapping image revealed the uniform deposition of the PEDOT:PSS layer on the TNP‐TCF substrate (bottom right of Figure 3b). Figure 3c shows the morphology of the TNP‐TCE, along with its schematic illustration. The PEDOT:PSS layer in the TNP‐TCE showed a pattern width of 940 µm with a separation gap of 120 µm, which was consistent with the line width of the EHD jet‐printed masks. The resulting TNP‐TCE exhibited high optical transparency (*T* = 87% at 550 nm wavelength), demonstrating the viability of the EHD jet printing process for fabricating micropatterned electrodes without impairing its optical transparency.

The widths of planar interdigitated electrodes are known to affect their electrochemical performance and optical transparency.^[^ ^56^, ^57^ ^]^ A series of TNP‐TCEs with different widths (3.75, 1.88, 1.25, and 0.94 mm, denoted as NX, where *X* represents the number of fingers (i.e., *X* = 4, 8, 12, and 16, respectively)) were prepared using the aforementioned fabrication technique (Figure S13a,b, Supporting Information). The Nyquist plots of the TNP‐TCEs with different electrode widths revealed that the cell resistance tended to decrease (0.83 (N4) → 0.43 kΩ (N16)) with decreasing the electrode width (Figure S13c, Supporting Information), indicating that the electrochemical performance enhanced with a decrease in the electrode width. In addition, the optical transparency of the TNP‐TCEs increased from 83 (N4) to 87% (N16) as the electrode width decreased (Figure S13d, Supporting Information). Meanwhile, a TNP‐TCE with precisely defined micro‐patterns could not be obtained with a further decrease in the electrode width below 0.94 mm (i.e., N16) because of the structural instability of EHD jet‐printed masks. These results indicated that among the fabricated TNP‐TCEs, the N16 exhibited the optimal interdigitated pattern. Figure 3e shows that a leaf beneath the TNP‐TCE (N16) can be clearly observed over a wide area, demonstrating the high optical transparency of the TNP‐TCE.

The interfacial adhesion between the PEDOT:PSS layer and underlying transparent substrate (i.e., TNP‐TCF) in the TNP‐TCE was examined by conducting a peel‐off test using a scotch tape. Compared to that of the control PET‐TCE (PEDOT:PSS on the AgNW‐deposited PET substrate), the PEDOT:PSS layer strongly adhered to the TNP‐TCF after the peel‐off test (Figure 3f). In addition, the peel strength (0.082 N mm^−1^) of the TNP‐TCE was remarkably higher than that (0.007 N mm^−1^) of the control electrode (Figure S14, Supporting Information). The superior interfacial adhesion of the TNP‐TCE was attributed to the intermolecular hydrogen bonding^[^ ^58^, ^59^ ^]^ between the hydroxyl/carbonyl groups of the TNP substrate and sulfonic acid (SO_3_H) groups of the PSS in the PEDOT:PSS electrode layer. To further elucidate this interfacial adhesion behavior of the TNP‐TCE, the depth profiles of its elements in the through‐thickness direction were analyzed. Compared to the control PET‐TCE, which showed an abrupt change in the concentration of S, a gradual decrease in S was observed at the interface between the PEDOT:PSS (top layer) and TNP‐TCF (bottom layer) of the TNP‐TCE (Figure 3g). This result was confirmed using the finite element method (FEM). The TNP‐TCE mitigated the in‐plane deformation of its ligaments and experienced the localized stress predominantly at the deformed zones, which constrained the out‐of‐plane deformation (Figure 3h) that resulted in a “stiffening effect”.^[^ ^13^ ^]^ In contrast, the control electrode collectively suffered from severe out‐of‐plane deformation and delamination.

### 2.4 Flexible TNP‐MSCs with On‐Demand Cell Configurations

**Figure 4:**
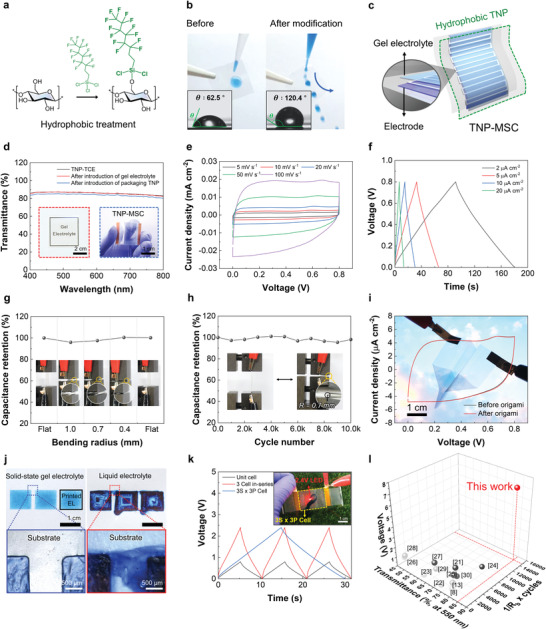
Flexible TNP‐MSCs with on‐demand cell configurations. a) Synthetic scheme of the hydrophobic silane (1H,1H,2H,2H‐perfluoro‐decyltrichlorosilane)‐treated TNP. b) Photographs of the water droplets on the pristine TNP (left) and hydrophobic silane‐treated TNP (right). Insets are the optical images of the water contact angle before and after the hydrophobic treatment. c) Schematic representation of the TNP‐MSC (composed of the microscale planar interdigitated TNP‐TCE, solid‐state gel electrolyte, and hydrophobic TNP packaging). d) Optical transmittance of the solid electrolyte‐deposited TNP‐TCE and TNP‐MSC in the wavelength range of 400–800 nm (visible light). Insets are their photographs. e) CV curves (measured at a scan rate of 5–100 mV s^−1^) of the TNP‐MSC. (f) GCD profiles of the TNP‐MSC over a wide range of areal current densities from 2–20 µA cm^−2^. g) Change in the specific capacitance of the TNP‐MSC as a function of the bending radius (*R*_b_). h) Capacitance retention (measured at a scan rate of 20 mV s^−1^) of the TNP‐MSC as a function of the cycle number under the bending deformation (at *R*_b_ = 0.7 mm). i) CV curves (measured at a scan rate of 50 mV s^−1^) and photograph (inset) of the TNP‐MSC under the origami airplane‐shaped state. j) OM images of the pixel arrays (area of the pixel = 1.0 × 1.0 cm^2^) deposited using the solid‐state gel electrolyte (left) and liquid electrolyte (right). k) GCD profiles of the TNP‐MSC (9 unit cells connected in‐series (3S) and in‐parallel (3P)) with a combined configuration of 3S × 3P. Inset is a photograph of a LED lamp powered by the TNP‐MSC. (l) Comparison of the optical transparency, operating voltage, and mechanical flexibility (expressed as *R*_b_^−1^ × bending cycles) of the TNP‐MSC to those of previously reported transparent MSCs.

In addition to the TNP‐TCEs, a hydrophobic silane‐treated TNP was prepared as a water‐proof packaging substance for fabricating nanocellulose transparent power sources. Briefly, hydroxyl groups of CNFs in the TNP were reacted with 1H,1H,2H,2H‐perfluoro‐decyltrichlorosilane^[^ ^60^ ^]^ to produce a hydrophobic layer (**Figure** **4**a). The obtained hydrophobic layer was verified by detecting the uniform distribution of fluorine (F) elements in the TOF‐SIMS 2D mapping image (Figure S15, Supporting Information). The water contact angle of the silane‐treated TNP was increased from 62.5 (of the pristine TNP) to 120.4°, which was sufficiently hydrophobic to repel water droplets (Figure 4b and Movie S2, Supporting Information).

A transparent solid‐state gel electrolyte (consisting of polyvinyl alcohol (PVA) matrix and 2.35 M lithium chloride (LiCl) aqueous electrolyte) was deposited on the aforementioned TNP‐TCE (thickness ≈23 µm) using the direct ink writing (DIW) process (Figure 4c). The transparent solid‐state gel electrolyte showed a good ionic conductivity of 2.1 mS cm^−1^ at room temperature (Figure S16, Supporting Information). In addition, owing to the PVA matrix that enabled intermolecular hydrogen bonding with the TNP substrate, the transparent gel electrolyte strongly adhered to the TNP‐TCE compared to the control electrode (PET‐TCE) (Figure S17, Supporting Information). Furthermore, the introduction of the solid‐state gel electrolyte and subsequent hydrophobic silane‐treated TNP packaging had no effect on the optical transparency of the resulting TNP‐MSC (Figure 4d).

The TNP‐MSC showed the typical pseudo‐capacitive charge/discharge behavior in the cyclic voltammetry (CV) curves (Figure 4e) and normal symmetric galvanostatic charge/discharge (GCD) profiles (Figure 4f), which appeared comparable to those of previous studies^[^ ^14^, ^17^ ^]^ on conducting polymers‐based MSCs. In addition, the TNP–MSC stably maintained its capacitance over 6000 charge/discharge cycles (Figure S18, Supporting Information).

The mechanical flexibility of the TNP‐MSC under various deformation modes was investigated. The TNP‐MSC maintained its capacitance at various *R*_b_ (from 1.0 to 0.4 mm) without losing its optical transparency (Figure 4g). In addition, the TNP‐MSC retained its capacitance (98% of the initial value) after 10 000 bending cycles (Figure 4h), which exceeded those of previously reported transparent MSCs (Table S2, Supporting Information). To underscore the mechanical flexibility of the TNP‐MSC, an airplane‐shaped TNP‐MSC cell was fabricated using an origami folding technique (Movie S3, Supporting Information). The obtained origami airplane‐shaped TNP‐MSC cell showed capacitance of 0.24 mF cm^−2^ and a stable CV curve (Figure 4i). In addition, no significant difference was observed in the pseudo‐capacitive behavior and optical transparency before and after the origami folding. This outstanding mechanical deformability of the TNP‐MSC was attributed to its elaborately designed multilayer interfaces and mechanical compliance of the nanocellulose paper.

The combination of the solid‐state gel electrolyte with the EHD jet‐printed micropatterned electrodes was expected to enable on‐demand (in‐series/in‐parallel) cell configurations with tunable voltages and capacitances in a single body of the TNP‐MSC, which is difficult to achieve with the traditional electrical connection of independently existing MSCs. To address this, unit MSC cells should be electrochemically isolated, exhibiting the essential use of solid‐state electrolytes.^[^ ^33^ ^]^ As a model experiment, solid‐state gel electrolyte was DIW‐printed exclusively on individual pixel electrodes (area of the pixel = 1.0 × 1.0 cm^2^) of the TNP‐TCEs without being connected, in which a blue dye (indigo carmine) was incorporated in the gel electrolyte for easy detection. Figure 4J shows that the solid‐state gel electrolyte on each pixel maintained its shape and prevented ionic contact between the adjacent pixels (separated by a gap of 1.0 mm) even after being subjected to the mechanical vibration. In contrast, the blue dye‐containing liquid electrolyte (2.35 M LiCl in water) showed severe flooding and crossover between the pixels, indicating that the liquid electrolyte was not suitable for maintaining the electrochemical isolation between the pixel electrodes due to its fluidic characteristic.

To explore the practical feasibility of the on‐demand (in‐series/in‐parallel) cell configuration, 9 unit cells of TNP‐MSC were connected in‐series (3S) and in‐parallel (3P) to obtain a combined configuration of 3S × 3P. The resulting TNP‐MSC showed a three‐fold increase in the voltage and capacitance (Figure 4k and Figure S19, Supporting Information). In addition, the TNP‐MSC (3S × 3P) successfully operated a LED lamp that requires an operating voltage of 2.4 V (inset of Figure 4k), exhibiting the electrical viability of the in‐series connection. More notably, the TNP‐ MSCs achieved a voltage of 7.2 V through the in‐series connection of the 9 unit cells (Figures S20 and S21, Supporting Information). These results demonstrated that the TNP‐MSC with the on‐demand cell configuration can act as a promising power source for optoelectronic devices requiring a wide range of operating voltages.

Furthermore, a single body of the TNP‐MSC with the in‐series connection of 3 unit cells stably maintained its capacitance under various folding modes (Figure S22, Supporting Information). To highlight the superior performances of the TNP‐MSC, a quantitative comparison with previously reported transparent MSCs was conducted, with focus on the operating voltage (7.2 V per cell), areal capacitance (0.24 mF cm^−2^), areal energy density (0.22 µWh cm^−2^), optical transparency (*T* = 85%), and mechanical flexibility (10000 bending cycles at *R*_b_ = 0.7 mm) (Figure 4l and Table S2, Supporting Information).

## 3 Conclusions

We have presented the transparent nanocellulose paper MSC (TNP‐MSC) as a natural material strategy to overcome the longstanding challenges of synthetic chemicals‐based transparent power sources. The transparent conductive film (TNP‐TCF), which consisted of an AgNW conductive layer on a thiol‐functionalized, semi‐IPN TNP substrate, exhibited the enhanced mechanical flexibility and thermal tolerance compared to the control PET‐TCF. The introduction of the EHD jet‐printed microscale polymeric masks on the TNP‐TCF enabled the fabrication of the PEDOT:PSS‐based transparent conductive electrode (TNP‐TCE) without impairing the optical transparency. Combined with a solid‐state gel electrolyte that could ionically isolate unit cells, the TNP‐TCE with a microscale planar interdigitated structure enabled on‐demand (in‐series/in‐parallel) cell configurations with tunable voltages and capacitances in a single body of TNP‐MSC. The resulting TNP‐MSC exhibited improvements in controllable voltage (7.2 V per cell), and mechanical flexibility (bending, multiple folding, and even origami airplane), while maintaining its optical transparency (*T* = 85%), which far exceeded those of previously reported transparent synthetic chemicals‐based transparent MSCs. We believe that the nanocellulose‐based transparent MSC strategy presented herein is a promising eco‐friendly and versatile platform technology that can be applied to next‐generation transparent power sources based on Li, Zn, and other electrochemical systems.

## 4 Experimental Section

### Design and Preparation of the Semi‐IPN TNP

2,2,6,6‐Tetramethylpiperidin‐1‐oxyl radical (TEMPO)‐mediated oxidized cellulose suspension (solid content = 1.0 wt.%, University of Maine) was diluted with deionized water to achieve a concentration of 0.35 wt.%. The diluted CNF dispersion (dry weight of CNFs = 0.21 g) was mixed with N‐hydroxyethyl acrylamide (HEAA) (20 wt.% of dry CNFs) using an ultra‐sonication to obtain a homogeneous mixture. Into the mixture, N,N’‐methylenebis(acrylamide) (MBAA) as a crosslinker (1.25 wt.% of HEAA) and ammonium persulfate as a photoinitiator (1.0 wt.% of HEAA) were added and then cast in a polypropylene petri dish followed by drying at room temperature for 3 days. The obtained film was then photo‐crosslinked using a UV lamp (Hg UV lamp, peak intensity = 5000 mW cm^−2^, Lichtezen) to produce a semi‐IPN TNP. Subsequently, the semi‐IPN TNP was immersed in a solution of ethanol/H_2_O (95/5, w/w) mixture containing 2.5 wt.% 3‐mercaptopropyltrimethoxysilane (MPTMS) for 3 h to allow polycondensation reaction between hydroxyl (from the CNF of semi‐IPN TNP) and thiol (from the MTPTMS) groups, in which the pH of the solution was adjusted to be 3.5. The thiol‐functionalized semi‐IPN TNP was rinsed with acetone and dried at 100 °C for 30 min in an N_2_ atmosphere. Meanwhile, to prepare nanocellulose‐based water‐proof packaging substance, the TNP was treated with hydrophobic 1H,1H,2H,2H‐perfluoro‐decyltrichlorosilane for 1 h.

### Preparation of the TNP‐TCEs with Microscale Planar Interdigitated Structure

UV‐curable mask ink (a mixture of NOA68T (Norland Product, Inc.)/SiO_2_ (average particle size = 40 nm, Denka) = 80/20 (w/w)) was introduced on top of the thiol‐functionalized semi‐IPN TNP substrate using a multifunctional electrohydrodynamic (EHD) jet‐printing system (Enjet Inc.) and followed by UV irradiation, yielding the microscale mask with interdigitated array patterns. The electric field applied to the EHD printing nozzle (inner diameter = 100 µm) allowed the elongated streaming of the ink, in which a voltage of 0.85 kV was induced over a distance of 60 µm between the nozzle and TNP substrate. Meanwhile, to prepare the electrode ink, AgNW solution (1 wt.% concentration in isopropyl alcohol, Flexio) was diluted to a concentration of 0.1 wt.%. The electrode ink was prepared by mixing a commercial PEDOT:PSS solution (Clevios PH1000, Heraeus) with a solvent mixture (consisting of isopropyl alcohol (IPA), deionized water, and dimethyl sulfoxide (DMSO)). The PEDOT:PSS solution was filtered with 0.45 µm syringe, in which the final weight‐based composition ratio of the electrode ink was PEDOT:PSS/IPA/DI water/DMSO = 22.9/68.3/7.1/1.7 (w/w/w/w). The AgNW and PEDOT:PSS solutions were sequentially deposited onto the TNP substrate using an air‐spray coater (HANTECH Co., Ltd.) at a spraying pressure of 30 kPa. The distance between the spray nozzle and the TNP substrate was kept at 17 cm.

### Preparation of the Flexible TNP‐MSCs

To prepare a transparent gel electrolyte, a mixture of 1.0 g of poly(vinyl alcohol) (PVA) and 1.0 g of LiCl were dissolved in 10 g of deionized water. The obtained gel electrolyte solution was DIW‐printed on top of the above‐prepared TNP‐TCE and dried at room temperature. Subsequently, the gel electrolyte/TNP‐TCE assembly was hermetically sealed with the hydrophobic silane‐treated TNP substrate (thickness = 23 µm) using thermal pressing at 60 °C, eventually producing the TNP‐MSC.

### Characterization of the TNP‐MSCs

The UV‐curing reaction of the semi‐IPN TNP was examined using Fourier‐transform infrared spectroscopy (FT‐IR) spectrometry (Alpha Platinum ATR, Bruker). The oxygen permeability of the semi‐IPN TNP and PET film were measured by using oxygen permeability (OP) analyzer (OX‐TRAN 2/21, MOCON). A coefficient of thermal expansion (CTE) was evaluated by using a thermomechanical analysis (TMA) (402 F1/F3, NETZSCH instruments) for a sample size of 30 mm in length and 5 mm in width from room temperature to 150 °C. Dynamic mechanical analysis (DMA) profiles were collected using a Q800 (TA Instruments).

The surface and cross‐sectional morphologies of the EHD jet‐printed UV‐curable masks and the TNP‐MSC components were examined by field emission scanning electron microscopy (FE‐SEM, S‐4800, Hitachi) equipped with energy dispersive X‐ray spectroscopy (EDS). The water contact angle of the semi‐IPN TNP substrate was measured using a drop shape analyzer (Phoenix 300, SEO). The physical appearance of the cell components was characterized using an optical microscope (LV100ND, Nikon). The electrical resistance (*R*_S_) of the TNP‐TCFs was measured using a four‐point probe measurement (CMT‐SR1000N, Advanced Instrument Technology). The optical transmittance profiles of the TNP‐MSC components were recorded by UV–vis‐spectrophotometry (Cary 5000, Agilent Tech). The covered area ratios (CAR) of the AgNWs on the TNP substrate were determined by digital image processing using the software ImageJ. The chemical analysis of the surface functionalized TNP was conducted using time‐of‐flight secondary ion mass spectrometry (TOF‐SIMS) (TOF‐SIMS5, ION TOF). The X‐ray photoelectron spectroscopy (XPS) (ESCALAB 250XI, Thermo Scientific) was performed to characterize chemical components of the TNP surface. The mechanical flexibility of the TNP‐MSCs and their components were quantitatively measured using a universal tensile tester (DA‐01, Petrol LAB) under various bending modes. The stress evolution of the TNP‐TCE under bending stresses was simulated using finite element analysis (FEA) software in Autodesk Inventor, in which the values of the material parameters involved in the analysis were as follows: Semi‐IPN TNP (Young's modulus (*E*) = 2.1 GPa, Poisson's ratio (*ν*) = 0.3) and PET film (*E* = 3.5 GPa, *ν* = 0.37). The ionic conductivity of the solid‐state gel electrolyte was characterized by the AC impedance over the frequency range from 10^−3^ to 10^6^ Hz at room temperature. The electrochemical performance of the TNP‐MSCs was measured using a potentiostat/galvanostat (VSP‐300, Bio‐Logic) at various charge/discharge conditions.

## Conflict of Interest

The authors declare no conflict of interest.

## Author Contributions

S. W. K. and Y. H. L. designed all chemical/electrochemical experiments and analyzed the results. J. G. G. and W. J. Y. performed physicochemical characterization of CNFs and modified CNFs. S. W. K. prepared the manuscript. S. Y. L. revised the manuscript critically. All authors have given approval to the final version of the manuscript.

## Supporting information

Supporting Information

Supplemental Movie 1

Supplemental Movie 2

Supplemental Movie 3

## Data Availability

The data that support the findings of this study are available from the corresponding author upon reasonable request.
