# Supplementary material for: Transparent and Multi‐Foldable Nanocellulose Paper Microsupercapacitors
Source: Adv Sci (Weinh). 2022 Oct 18;9(34):2203720. doi: 10.1002/advs.202203720 (PMC9731695; doi:10.1002/advs.202203720)
Supplement: Supplementary file 1 — Supporting Information [file ADVS-9-2203720-s004.pdf]

ADVANCED SCIENCE
Open Access

Supporting Information

for *Adv. Sci.*, DOI 10.1002/advs.202203720

Transparent and Multi-Foldable Nanocellulose Paper Microsupercapacitors

*Sang-Woo Kim, Kwon-Hyung Lee, Yong-Hyeok Lee, Won-Jae Youe, Jae-Gyoung Gwon and Sang-Young Lee**

Supporting Information

Transparent and Multi-Foldable Nanocellulose Paper Microsupercapacitors

*Sang-Woo Kim, Kwon-Hyung Lee, Yong-Hyeok Lee, Won-Jae Youe, Jae-Gyoung Gwon, and Sang Young Lee**

**Abbreviation**	**Full name**
TNP	Transparent nanocellulose paper
TNP-TCF	Transparent nanocellulose paper-based transparent conductive film
TNP-TCE	Transparent nanocellulose paper-based transparent electrode
TNP-MSC	Transparent nanocellulose paper microsupercapacitors

List of keyword abbreviations

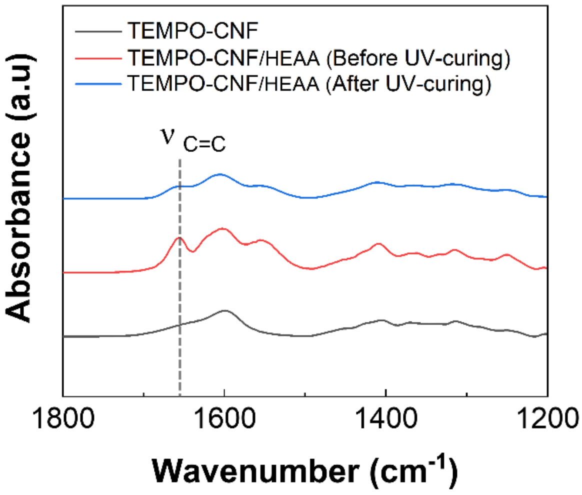

Figure S1. Change in the characteristic Fourier transform infrared (FT-IR) peaks assigned to the acrylic C═C bonds (1610–1625 cm^{-1}) of the 2,2,6,6-tetramethylpiperidin-1-oxyl-oxidized–cellulose nanofibers (TEMPO-CNF)/N-hydroxyethyl acrylamide (HEAA) mixture before and after the UV curing.

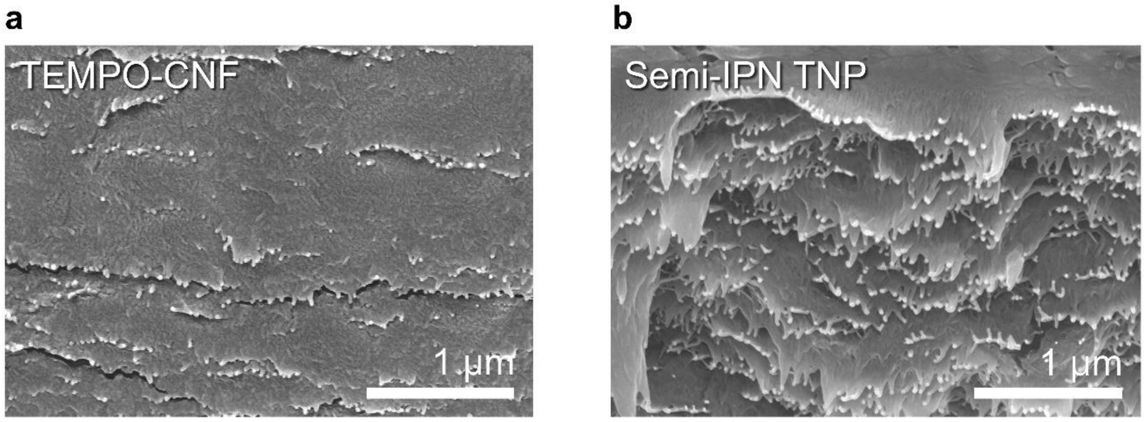

Figure S2. Cross-sectional scanning electron microscopy (SEM) images of the fractured (a) TEMPO-CNF film and (b) Semi-interpenetrating polymer network (IPN)-TNP after the tensile test.

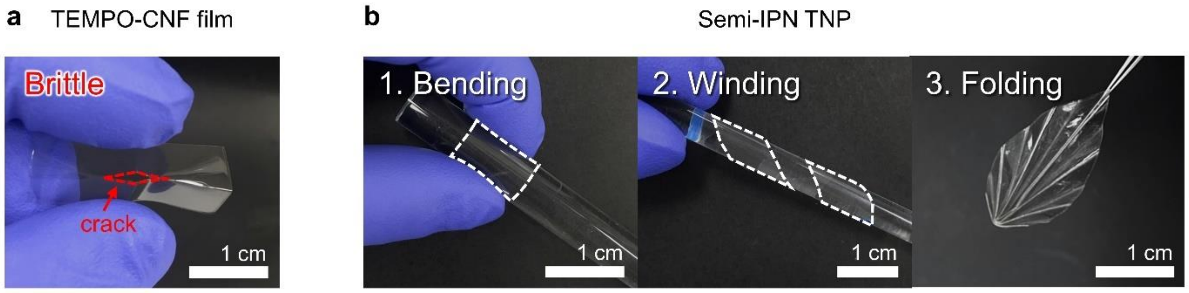

Figure S3. Photographs of (a) TEMPO-CNF film and (b) semi-IPN TNP under various deformation modes (bending (bending radius (R_b) = 5.0 mm), winding along a rod (radius = 5.0 mm), and multiple folding).

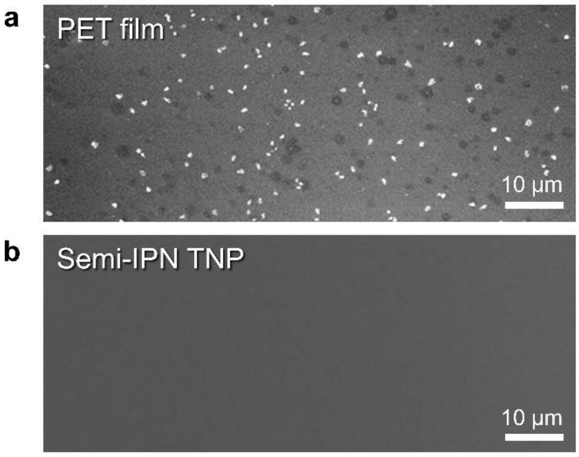

Figure S4. SEM images of (**a**) polyethylene terephthalate (PET) film (control) and (**b**) semi-IPN TNP after thermal treatment (at 140 °C for 2 h).

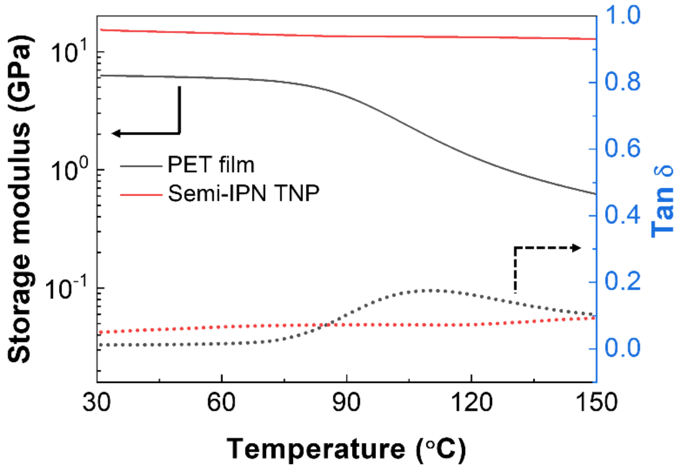

Figure S5. *Dynamic mechanical analysis (*DMA) profiles of the PET film (control) and the semi-IPN TNP as a function of temperature (at a heating rate of 5 °C min^{-1} under N_2 atmosphere).

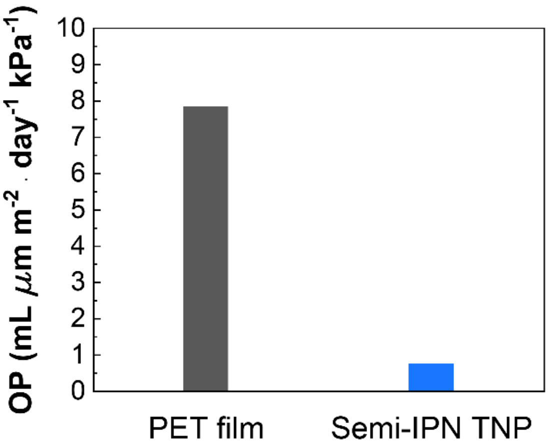

Figure S6. Oxygen barrier properties of the PET Film (control) and semi-IPN TNP (at 23 ± 1 °C and relative humidity of 0%).

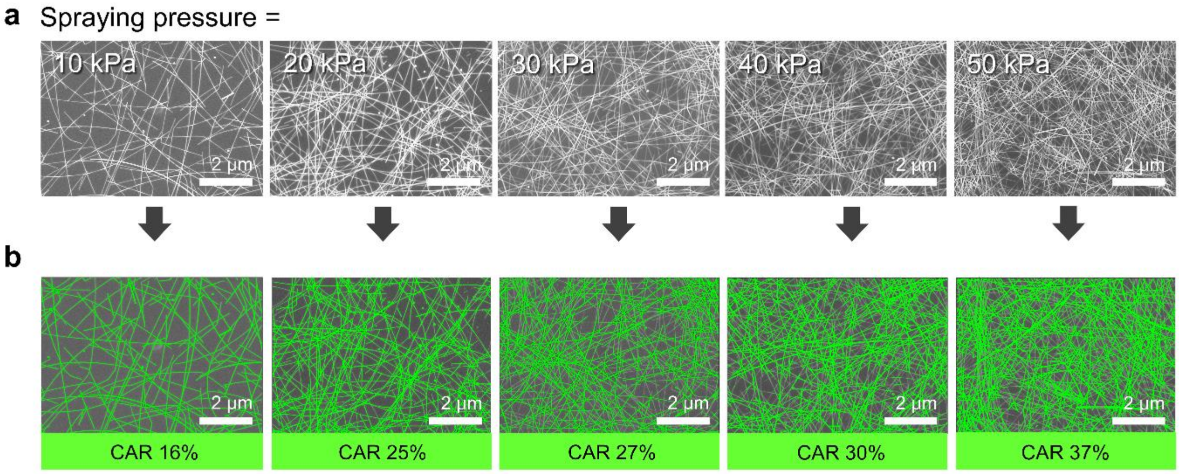

Figure S7. (a) SEM images of the TNP-TCFs as a function of the spraying pressure. (b) Converted images (*via* ImageJ) showing the projected silver nanowires (AgNWs) networks expressed based on the covered area ratio (CAR).

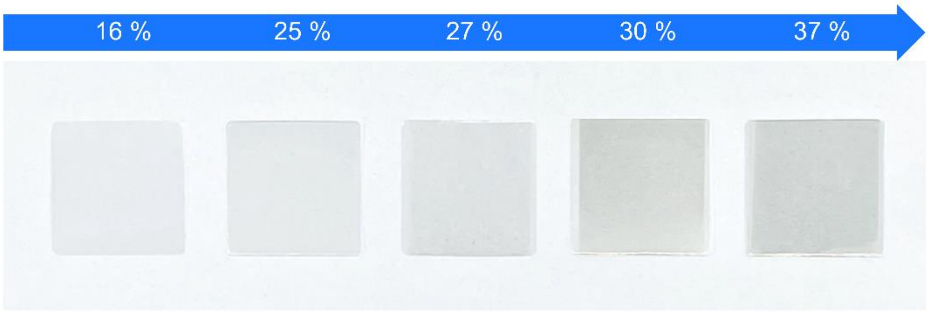

Figure S8. Photographs of the TNP-TCFs as a function of the CAR.

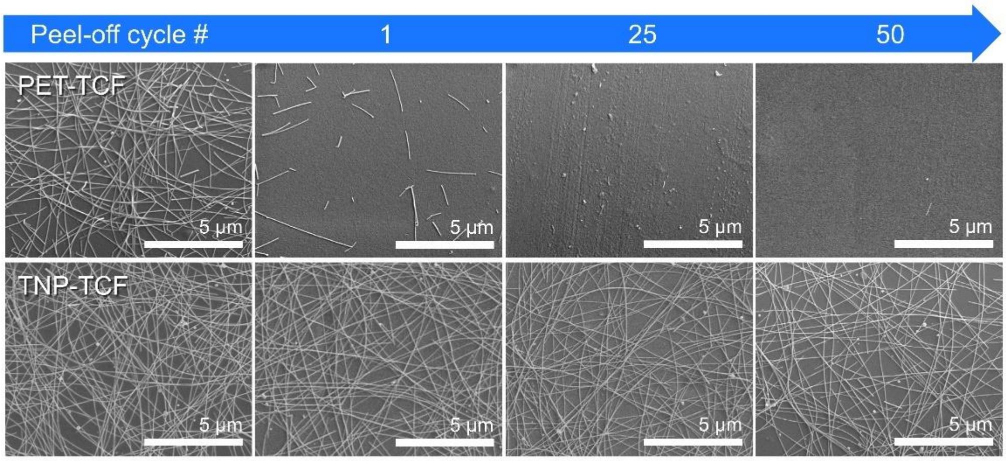

Figure S9. SEM images of the PET-TCF (control) and TNP-TCF after the peel-off test (at a peel-off speed of 5.0 mm min^{-1}) as a function of the peel-off cycle.

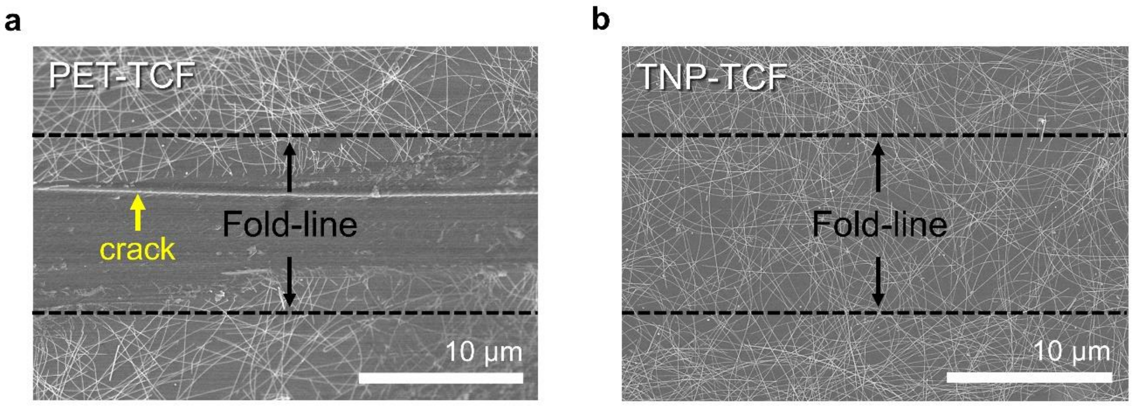

Figure S10. SEM images of (a) the PET-TCF (control) and (b) the TNP-TCF after the origami folding.

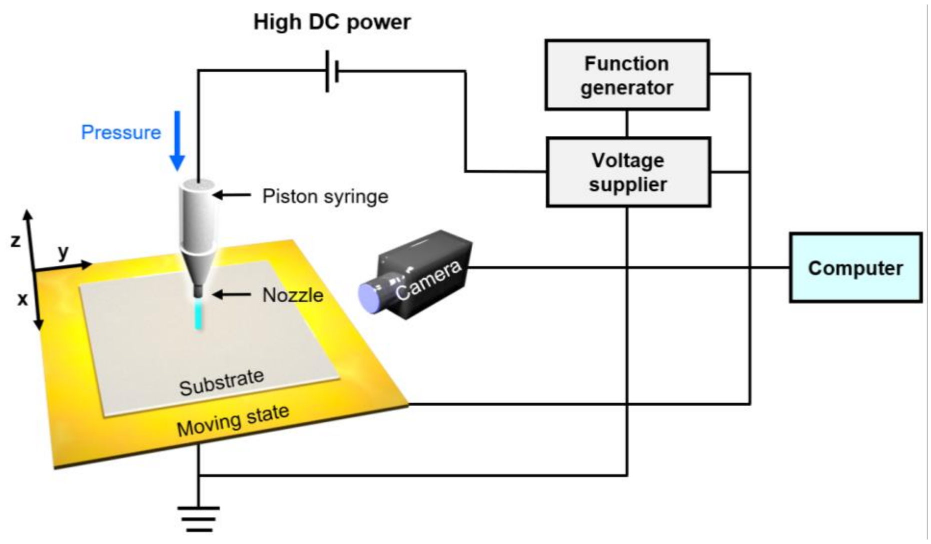

Figure S11. Schematic representation of the electrohydrodynamic (EHD) jet-printing equipment, which consists of a nozzle connected to a piston syringe, a camera set-up for observing the in-situ printing process, and a computer-controlled three-axis moving stage.

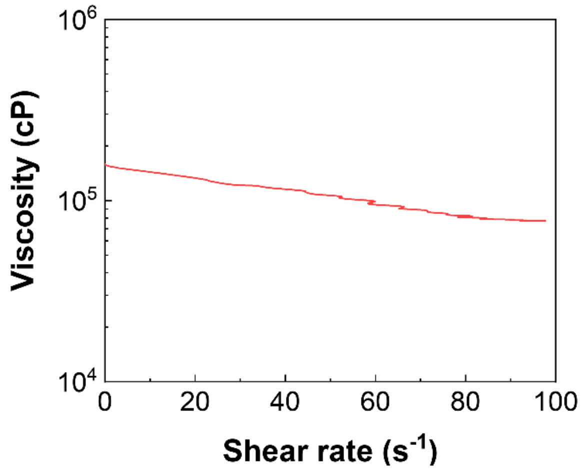

Figure S12. Viscosity of the UV-curable mask ink as a function of the shear rate.

a

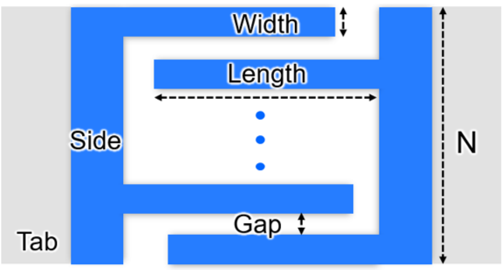

b

Name	N (ea)	Width (mm)	Gap (μm)	Side (mm)	Length (mm)
N4	4	3.75	120	2.5	10
N8	8	1.875	120	2.5	10
N12	12	1.25	120	2.5	10
N16	16	0.9375	120	2.5	10

c

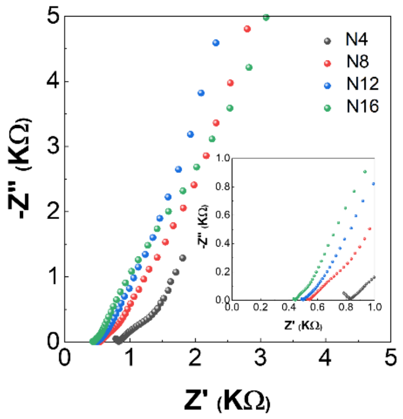

d

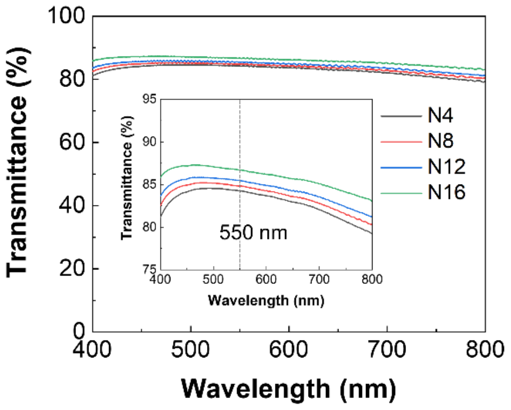

Figure S13. (a) Schematic representation describing the TNP-MSCs configuration. (b) Dimension values of the TNP-MSC configuration as a function of the number of fingers. (c) Nyquist plots, and (d) transmittance spectra in the visible range (400–800 nm) of the TNP-MSCs as a function of the number of fingers.

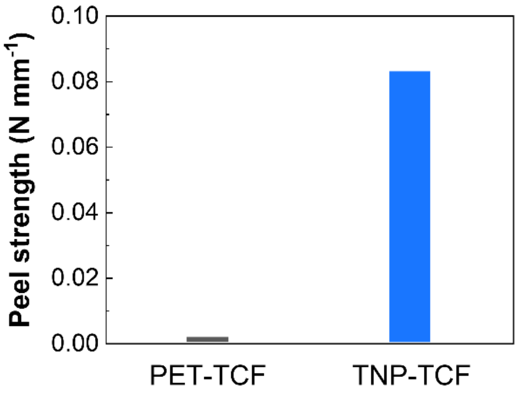

Figure 14. Peel strength of the interface between the poly(3,4-ethylene dioxythiophene):(styrene sulfonate) (PEDOT:PSS) layer and underlying transparent substrates at a peel-off speed of 5.0 mm min^{-1}: PET-TCF (control) vs. TNP-TCF.

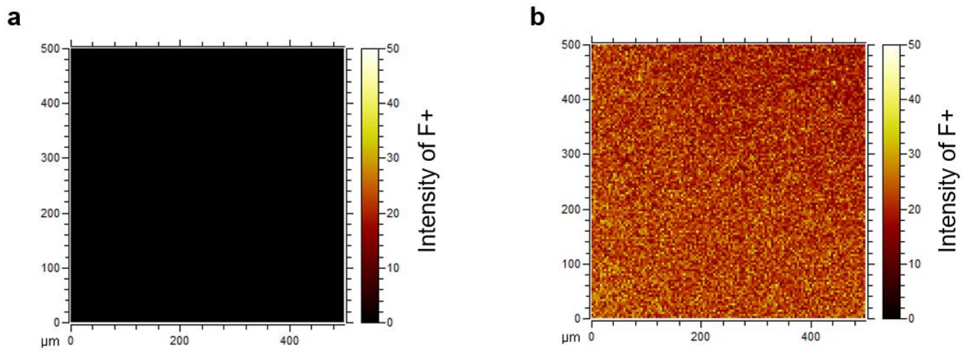

Figure S15. Time-of-flight secondary ion mass spectroscopy (TOF-SIMS) 2D mapping images of F^+ fragments. (a) Bare TNP film and (b) Hydrophobic silane-treated TNP film.

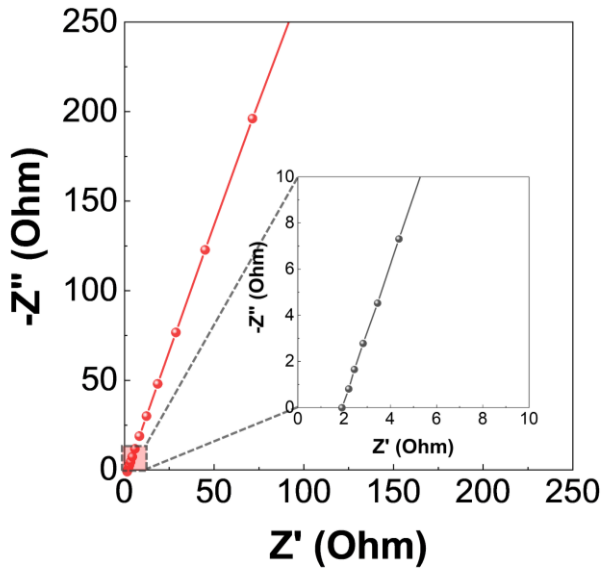

Figure S16. Nyquist plot of the transparent solid-state gel electrolyte (comprising of polyvinyl alcohol (PVA) matrix and 2.35 M lithium chloride (LiCl) aqueous electrolyte) at room temperature.

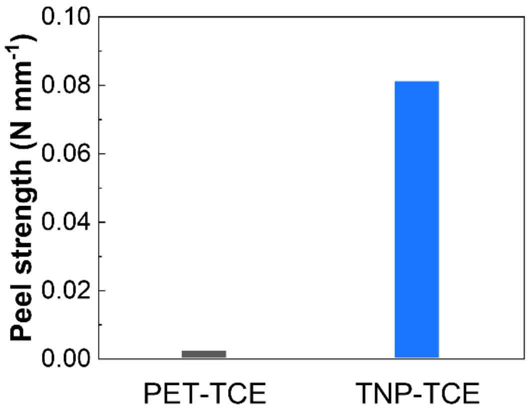

Figure S17. Peel strength of the interface between the solid-state gel electrolyte layer and TNP-TCE (vs. PET-TCE (control)).

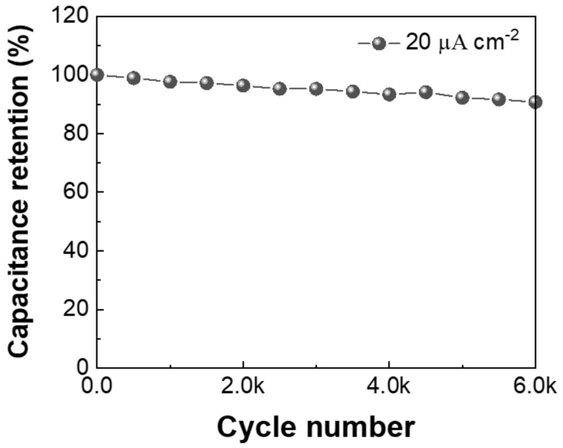

Figure S18. Capacitance retention (measured at areal current density of 20 μA cm^{-2}) of the TNP–MSC as a function of charge/discharge cycle number.

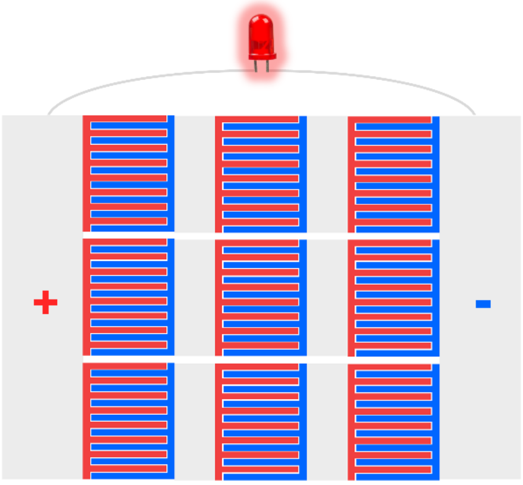

Figure S19. Schematic representation of the 9 unit cells with a combined configuration of 3S (3 cells in-series) × 3P (3 cells in-parallel).

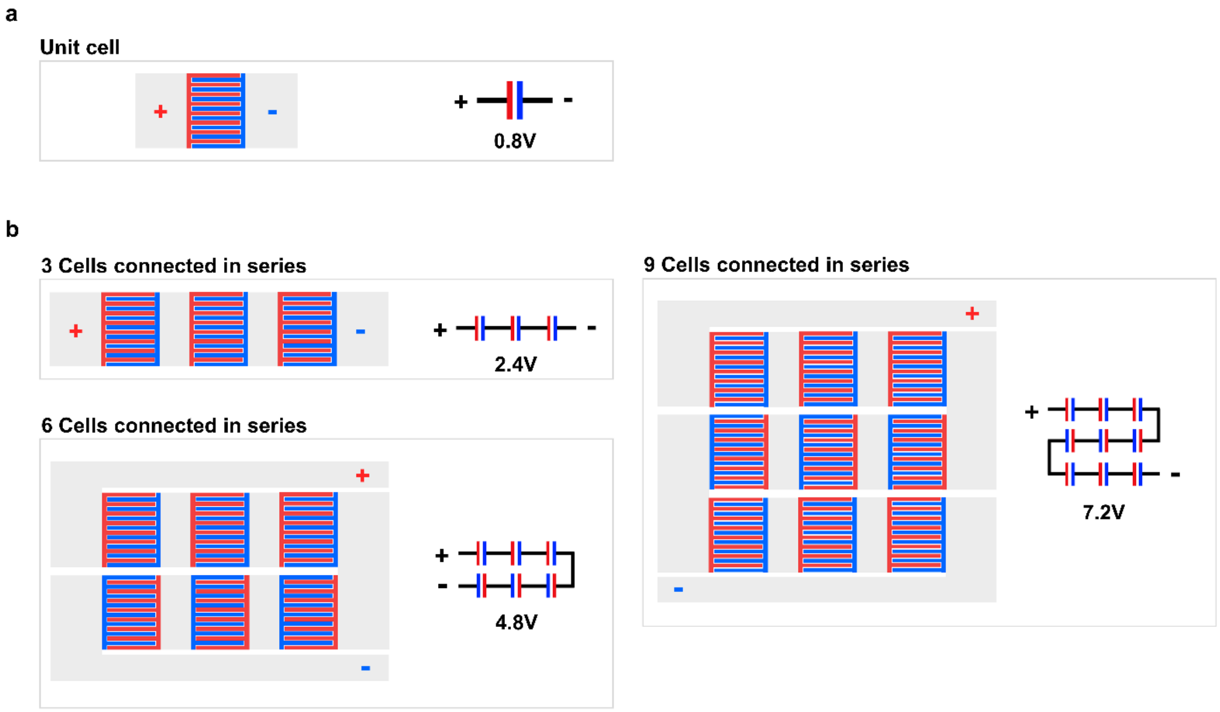

Figure S20. Schematic representation of the TNP-MSCs. (a) Unit Cell. (b) 3 cells, 6 cells, and 9 cells connected in-series.

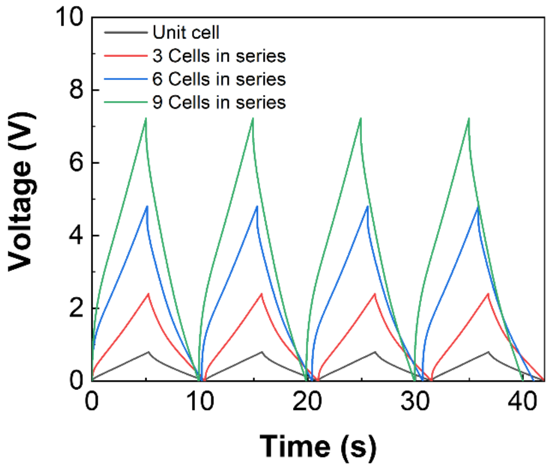

Figure S21. Galvanostatic charge/discharge **(**GCD) profiles of the TNP-MSCs connected in series (measured at areal current density of 20 μA cm^{-2}).

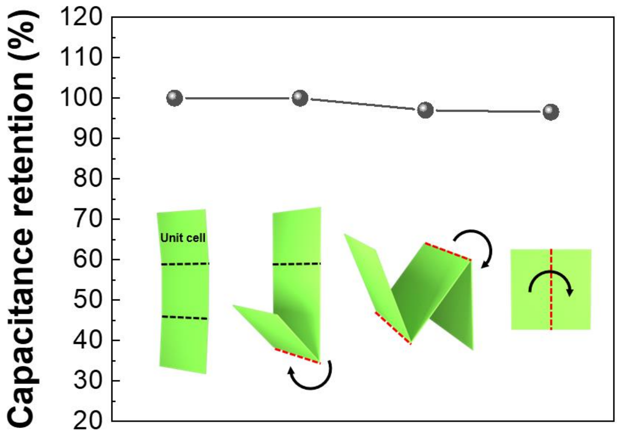

Figure S22. Capacitance retention (measured at a scan rate of 20 mV s^{-1}) of a single body of the TNP-MSC with in-series connection of 3 unit cells under various folding modes.

Table 1. Comparison of the optical transmittance, electrical conductivity, and mechanical flexibility of the TNP-TCF (this work) to those of previously reported TCFs based on synthetic polymer substrates.

Substrate	Conductive materials	Transmittance [%][a)]	Electrical conductivity [Ω sq^{-1}]	Bending radius [mm]	Flexibility [$\Delta R/R_0$][b)]	Ref.
Nanocellulose paper	AgNWs	7	31	0.5	Negligibly changed, after 10,000 cycles	This work
Nylon/cellulose acetate film	PANi	39	188	5	90%, after 1,000 cycles	[1]
Nanocellulose paper	AgNWs	82	54	2	Negligibly changed, 500 cycles	[2]
PDA@NFC-PEDOT:PSS@AgNW		92.6	7.32	5	Negligibly changed, 1,000 cycles	[3]
Nanocellulose paper	AgNW/ PEDOT:PSS	84	35	4	Negligibly changed, 400 cycles	[4]
Nanocellulose paper	AgNWs	81.4	40.3	4	Negligibly changed, 500 cycles	[5]
PET	PEDOT:PSS	84.6	24	3	Negligibly changed, after 5,000 cycles	[6]
PET	AgNFs	95	12	4	Negligibly changed, 1,000 cycles	[7]
PET	Ag grid	89.54	2	10	Negligibly changed, after 10,000 cycles	[8]
PET	SWCNT	91	40	5	Negligibly changed, after 10,000 cycles	[9]
PET	Au-Cu mesh	79	44	6	Increasing ~ 7%, after 10,000 cycles	[10]
PET	AgNWs/ZnO	93	41	3	Negligibly changed, after 1,000 cycles	[11]
PET	NiOx	84	53	10	Negligibly changed, after 10,000 cycles	[12]
PEN	Cu@Ni@NiCoS	89	12	3	Increasing ~ 5%, after 10,000 cycles	[13]
PEN	$Co(OH)_2$@Ni	87	25	3	Increasing ~ 10%, after 10,000 cycles	[14]
PET	AgNWs	78	50	5	Negligibly changed, after 10,000 cycles	[15]
PET	TiO2/Au/TiO_2 nanomesh	93.5	70	2.5	Negligibly changed, after 1,000 cycles	[16]
PDMS	Ag-AuNWs	63	15	5	Increasing ~ 0.2%, after 5,000 cycles	[17]
PDMS	Ag-AuNWs	88	50	5	Negligibly changed, after 5,000 cycles	[18]
PET	AgNPs	85.79 (0.75)	0.75	3	Negligible change, after 1,000 cycles	[19]
PET	Ti3C2Tx	87.8 (123)	123	3	Increasing 6%, after 1,000 cycles	[20]

[a)]Transmittance indicates the value at wavelength of 550 nm; [b)]Relative electrical resistance change ($\Delta R/R_0$) indicates the sheet resistance change before and after bending cycles.

Table 2. Comparison of the optical transmittance, mechanical flexibility, areal capacitance, areal energy density, and operating voltage of the TNP-MSC (this work) to those of previously reported transparent MSCs.

TCF	Active material	Transmittance [%][a)]	Bending radius [mm]	Areal capacitance [mF cm^{-2}]	Areal energy density [μWh cm^{-2}]	Flexibility [$\Delta C/C_0$][b)]	Operating voltage [V]	Ref.
AgNWs/nanocellulose paper	PEDOT:PSS	85	0.7	0.24	0.22	98%, 10,000 cycles Origami airplane	0.8–7.2	This work
rGO/nanocellulose paper	WO_3	83	15	-	-	99.1%, after 100 cycles	2.5	[21]
Graphene/PET	Graphene	75	12.5	0.1	0.01	86.5%, after 1,000 cycles	0.8	[22]
Ag/Au/PET	PPy	64	11	0.58	0.03	93%, after 1,000 cycles	0.8	[23]
Ag grid/PET	PEDOT:PSS	80.58	2	2.79	0.25	97.4%, after 1,000 cycles	0.8	[8]
AgNWs/NOA	PEDOT:PSS	80	1	3.3	0.3	90%, after 8,000 cycles	0.8	[24]
AgNFs/NOA	PEDOT:PSS	77.4	2	0.91	0.09	98.5%, after 5,000 cycles	1.0–2.0	[25]
Au/NOA	Graphene	48	2	0.08	0.006	87.5%, after 1,800 cycles	1	[26]
Ag grid/PET	$Ni_xFe_yO_z$@rGO	70.6	3	0.23	0.12	Almost unchanged, 1,000 cycles	0–2.5	[27]
Ni mesh/PEN	MnO_2	51	5	7.31	0.86	90%, 1,000 cycles	1.6	[28]
PET	PEDOT:PSS	65	2	4.7	0.21	Almost unchanged, 5,000 cycles	0.8	[29]
Cu@NiNF/PEN	NiCoS	65	3	0.01	0.48	95%, 10,000 cycles	0.8	[13]

Au/PET	MnO_2	81.7	5	1.33	0.06	90%,<br>1,000 cycles	0.8–1.6	[30]

[a)]Transmittance indicates the value at wavelength of 550 nm; [b)]Relative capacitance change ($\Delta C/C_0$) indicates the areal capacitance change before and after bending cycles.

Supplementary notes

The areal capacitance (C_A) and areal energy density (E_A) of the TNP-MSC were calculated using the following equation (1) and (2):

$$C_A = \frac{\Delta Q}{\Delta V \times \Pi} = \frac{\int_{V_1}^{V_2} i dV}{\Delta V \times v \times \Pi} \qquad (1)$$

$$E_A = \frac{C \times \Delta V^2}{2 \times 3600 \times \Pi} \qquad (2)$$

where i is the current, ΔV is the voltage window, v is the scan rate, C is the specific capacitance, and Π is the total area of the TNP-MSC.

Movie S1. Video clip showing the EHD printing of the UV-curable mask ink on the semi-IPN TNP substrate.

Movie S2. Video clip showing the rolling-off of water droplets on the tilted substrates: pristine TNP (control) vs. hydrophobic silane-treated TNP.

Movie S3. Video clip showing the folding/unfolding of the origami airplane-shaped TNP-MSC.

References

[1] K. Devarayan, D. Lei, H.-Y. Kim, B.-S. Kim, *Chem. Eng. J.* **2015**, 273, 603–609.

[2] D. Kim, Y. Ko, G. Kwon, U.-J. Kim, J. You, *Appl. Mater. Interfaces.* **2018**, 10, 38517−38525.

[3] Y. Su, S. Yuan, S. Cao, M. Miao, L. Shi, X. Feng, *J. Mater. Chem. C* **2019**, *7*, 14123−14129.

[4] X. Wang, J. Zhou, Y. Zhu, W. Cheng, D. Zhao, G. Xu, H. Yu, *Chem. Eng. J.* **2020**, 392, 123644.

[5] Y. Wang, J.-T. Huang, *RSC Adv.* **2021**, 11, 36607-36616.

[6] X. Hu, X. Meng, L. Zhang, Y. Zhang, Z. Cai, Z. Huang, M. Su, Y. Wang, M. Li, F. Li, *Joule* **2019**, 3, 2205-2218.

[7] S. Lin, X. Bai, H. Wang, H. Wang, J. Song, K. Huang, C. Wang, N. Wang, B. Li, M. Lei, *Adv. Mater.* **2017**, 29, 1703238.

[8] J.-L. Xu, Y.-H. Liu, X. Gao, Y. Sun, S. Shen, X. Cai, L. Chen, S.-D. Wang, *Appl. Mater. Interfaces*. **2017**, 9, 27649−27656.

[9] S. Jiang, P.-X. Hou, M.-L. Chen, B.-W. Wang, D.-M. Sun, D.-M. Tang, Q. Jin, Q.-X. Guo, D.-D. Zhang, J.-H. Du, *Sci. Adv.* **2018**, 4, eaap9264.

[10] A. Zumeit, A. S. Dahiya, A. Christou, D. Shakthivel, R. Dahiya, *npj Flex. Electron* **2021**, 5, 1–10.

[11] Y.-X. Zhang, J. Fang, W. Li, Y. Shen, J.-D. Chen, Y. Li, H. Gu, S. Pelivani, M. Zhang, Y. Li, *ACS Nano* **2019**, 13, 4686−4694.

[12] V. B. Nam, J. Shin, Y. Yoon, T. T. Giang, J. Kwon, Y. D. Suh, J. Yeo, S. Hong, S. H. Ko, D. Lee, *Adv. Funct. Mater*. **2019**, 29, 1806895.

[13] B. S. Soram, I. S. Thangjam, J. Y. Dai, T. Kshetri, N. H. Kim, J. H. Lee, *Chem. Eng. J.* **2020**, 395, 125019.

[14] B. S. Soram, J. Dai, T. Kshetri, N. H. Kim, J. H. Lee, *Chem. Eng. J.* **2020**, 391, 123540.

[15] S. Huang, Q. Zhang, F. Yang, D. T. Gangadharan, P. Li, F. Ren, B. Sun, D. Ma, *J. Mater. Chem. A* **2020**, 8, 8620–8628.

[16] T. Qiu, B. Luo, E. M. Akinoglu, J. H. Yun, I. R. Gentle, L. Wang, *Adv. Funct. Mater.* **2020**, 30, 2002556.

[17] W. Y. Jin, M. M. Ovhal, H. B. Lee, B. Tyagi, J. W. Kang, *Adv. Energy Mater.* **2021**, 11, 2003509.

[18] S. Huang, Y. Liu, M. Jafari, M. Siaj, H. Wang, S. Xiao, D. Ma , *Adv. Funct. Mater.* **2021**, 31, 2010022.

[19] X. Zhu, M. Liu, X. Qi, H. Li, Y. F. Zhang, Z. Li, Z. Peng, J. Yang, L. Qian, Q. Xu, *Adv. Mater.* **2021**, 33, 2007772.

[20] R. Li, X. Ma, J. Li, J. Cao, H. Gao, T. Li, X. Zhang, L. Wang, Q. Zhang, G. Wang, *Nat. Commun.* **2021**, 12, 1587.

[21] T. G. Yun, D. Kim, Y. H. Kim, M. Park, S. Hyun, S. M. Han, *Adv. Mater*. **2017**, 29, 1606728.

[22] K. Jo, S. Lee, S.-M. Kim, J. B. In, S.-M. Lee, J.-H. Kim, H.-J. Lee, K.-S. Kim, *Chem. Mater*. **2015**, 27, 3621–3627.

[23] H. Moon, H. Lee, J. Kwon, Y. D. Suh, D. K. Kim, I. Ha, J. Yeo, S. Hong, S. H. Ko, *Sci. Rep.* **2017**, 7, 41981.

[24] R. T. Ginting, M. M. Ovhal, J.-W. Kang, *Nano energy* **2018**, 53, 650–657.

[25] S. B. Singh, T. Kshetri, T. I. Singh, N. H. Kim, J. H. Lee, *Chem. Eng. J.* **2019**, 359, 197–207.

[26] Y. Chen, X.-Y. Fu, Y.-Y. Yue, N. Zhang, J. Feng, H.-B. Sun, *Appl. Surf. Sci.* **2019**, 467, 104–111.

[27] T. Liu, R. Yan, H. Huang, L. Pan, X. Cao, A. DeMello, M. Niederberger, *Adv. Funct. Mater.* **2020**, 30, 2004410.

[28] B. S. Soram, J. Y. Dai, I. S. Thangjam, N. H. Kim, J. H. Lee, *J. Mater. Chem. A* **2020**, 8, 24040–24052.

[29] M. M. Ovhal, N. Kumar, S. Lim, J.-W. Kang, *Appl. Surf. Sci.* **2020**, 529, 147072.

[30] J.-P. Chen, W. Huang, Z.-Y. Jiang, J.-L. Xu, S.-Q. Zhao, Y.-H. Liu, *J. Phys. D: Appl. Phys.* **2020**, 53, 165501.
